# Evidence for reduced synaptic protein SNAP-25 in cerebrospinal fluid in major depressive disorder and schizophrenia

**DOI:** 10.1136/bmjment-2025-301752

**Published:** 2025-08-21

**Authors:** Petra Steinacker, Leonie Werner, Alexander Tarabuko, Ilyas Al-Ali, Naguib Mechawar, Christopher R Pryce, Nadia Cattane, Giulia Poggi, MHD Rami Al Shweiki, Heiko Graf, Henning Großkopf, Steffen Halbgebauer, Patrick Oeckl, Lorenzo Barba, Laura Meier, Samir Abu-Rumeileh, Hugh Marston, Klaus D Bornemann, Bastian Hengerer, Karin M Danzer, Carlos Schönfeldt-Lecuona, Markus Otto

**Affiliations:** 1 Department of Neurology, Martin Luther University Halle Wittenberg, Halle (Saale), Germany; 2 Department of Neurology, Ulm University Hospital, Ulm, Germany; 3 Department of Psychiatry, McGill University, Montreal, Quebec, Canada; 4 University Hospital of Psychiatry, University of Zurich, Zurich, Switzerland; 5 Biological Psychiatry Unit, IRCCS Istituto Centro San Giovanni di Dio Fatebenefratelli, Brescia, Italy; 6 Department of Psychiatry and Psychotherapy III, Ulm University Hospital, Ulm, Germany; 7 DZNE Standort Ulm, Ulm, Germany; 8 CNS Diseases Research, Boehringer Ingelheim Pharma GmbH and Co. KG, Biberach an der Riss, Germany

**Keywords:** Depression, Cross-Sectional Studies

## Abstract

**Background:**

Decreased cerebrospinal fluid (CSF) levels of synaptic proteins, possibly reflecting impaired synaptic function, have been observed in major depressive disorder (MDD).

**Objective:**

To investigate the diagnostic utility of the soluble N-ethylmaleimide-sensitive-factor attachment receptor (SNARE) complex protein, synaptosomal-associated protein of 25 kDa (SNAP-25), for MDD.

**Methods:**

Overall, 208 participants with one of MDD, schizophrenia (SCZ) or bipolar disorder (BD), and healthy controls (HCs) were retrospectively enrolled. CSF levels of SNAP-25 were assessed relative to MDD characteristics and the diagnostic potential was analysed. In subgroups of patients, CSF levels of presynaptic neurexin 3 (NRXN3), postsynaptic neurogranin (NRGN) and Alzheimer’s disease biomarkers were measured for comparison.

**Findings:**

SNAP-25 levels, but not the levels of the other synaptic markers, were significantly decreased in MDD compared with HCs, allowing for discrimination with 68% sensitivity and 67% specificity. SNAP-25 was not associated with MDD severity or antidepressant medication. Compared with HCs, SCZ also displayed decreased SNAP-25 enabling discrimination with 64% sensitivity and 77% specificity. There were strong correlations between levels of synaptic proteins and established Alzheimer pathology markers, with subtle differences in the association pattern between disorders.

**Discussion:**

Our data suggest that SNAP-25, NRXN3 and NRGN versus beta-amyloid and phosphorylated tau protein 181 (ptau) are regulated differentially across psychiatric disorders and that SNAP-25 has a moderate diagnostic potential for MDD and SCZ. We propose that CSF SNAP-25 level might represent an integrated readout of reduced synaptic function, rather than of synaptic degeneration, in MDD. Further studies are needed to analyse whether this potential can be increased by using multimarker measurements and whether it will be possible to subtype psychiatric disorders according to synaptic involvement in pathophysiology.

**Clinical implications:**

SNAP-25 and other synaptic proteins in CSF might aid diagnosis and subtyping of MDD and SCZ. The current development of sensitive methods to also determine synaptic proteins in blood samples from patients will advance the validation of the biomarker potential and contribute to understanding of synaptic involvement in the pathophysiology of MDD and SCZ.

**WHAT IS ALREADY KNOWN ON THIS TOPIC**

- Levels of some synaptic proteins are decreased in cerebrospinal fluid of patients with major depressive disorder (MDD), potentially reflecting synaptic dysfunction.

**WHAT THIS STUDY ADDS**

- Reduced synaptosomal-associated protein of 25 kDa (SNAP-25) has a moderate diagnostic potential for MDD and schizophrenia (SCZ). We propose that cerebrospinal fluid (CSF) SNAP-25 level might represent an integrated readout of reduced synaptic function, rather than of synaptic degeneration, in MDD and SCZ.
- Furthermore, synaptic and Alzheimer pathology markers correlate in MDD, SCZ and bipolar disorder, with subtle differences between disorders.

**HOW THIS STUDY MIGHT AFFECT RESEARCH, PRACTICE OR POLICY**

- SNAP-25 in CSF has potential as a diagnostic marker in MDD and SCZ, and can contribute to understanding the role of altered synaptic function in the pathogenesis of these disorders. Future studies will show if multimarker measurements increase the diagnostic potential and support subtyping of psychiatric disorders according to synaptic involvement in pathophysiology.

## Introduction

 Worldwide, depression is the third leading cause of years lived with disability.^1^ Major depressive disorder (MDD) is characterised by persistently depressed mood and/or loss of interest and is diagnosed on the persistent manifestation of these and a group of additional symptoms. Despite the generally promising treatment options, MDD is characterised by recurrent depressive episodes, with approximately 30% being classified as treatment-resistant and 15% developing chronic depression.^2^ The diagnosis of depression can be challenging as there is a notable overlap of symptoms with other psychiatric disorders such as schizophrenia (SCZ) or bipolar disorder (BD). Furthermore, emotional symptoms might also occur in other conditions such as neurodegenerative diseases.^3^ Currently, for MDD, there is no reliable cerebrospinal fluid (CSF) marker available to aid differential diagnosis, to subtype patients, to allow disease staging, or to prognose treatment response.

Disturbances in synaptic function are proposed as pathophysiological causes of MDD. For example, synaptic loss occurs in the dorsolateral prefrontal cortex,^4^ and impaired function and changes in synaptic plasticity were observed in cortical and subcortical circuits.^5^ Several molecular factors have been suggested to underlie the observed synaptic dysfunctions. Recent biomarker research focussing on diagnosis and monitoring of neurodegenerative diseases has demonstrated the relevance of synaptic biomarkers, especially early in the disease course and even presymptomatically. We hypothesised that synaptic protein levels could also be changed in primary psychiatric disorders and, indeed, compared with controls, attenuated expression levels, of for example, neurexin-3 (NRXN3), contactin-associated protein-like 4 (CNTNAP4) and glutamate ionotropic receptor AMPA type subunit 4 (GRIA4) were detected applying a proteomic approach.^6^

The present study was conducted to investigate our primary hypothesis that the CSF level of synaptosomal-associated protein 25 (SNAP-25), a presynaptic protein responsible for vesicle fusion as part of the soluble N-ethylmaleimide-sensitive-factor attachment receptor (SNARE) complex and a promising biomarker of synaptic function in neurodegenerative diseases,^7 8^ is of diagnostic utility for MDD. As diagnostic comparison groups, we included healthy controls (HCs) and patients with either BD or SCZ. Furthermore, we investigated a second hypothesis, that changes in synaptic proteins reflect disorder-specific pathological processes, and for this two further synaptic proteins were analysed, namely NRXN3, a presynaptic cell adhesion molecule involved in synapse formation and synaptic signalling and identified as downregulated in MDD as mentioned before,^6^ and neurogranin (NGRN), a postsynaptic protein regulated by synaptic activity and involved in plasticity. We also assessed the association of the synaptic proteins with established biomarkers of neurodegenerative disease pathology (beta-amyloid (Aβ) 1–42 and 1–40, ptau, total tau, neurofilament light chain (NfL)) to study aetiopathogenic aspects.

## Methods

### Participants

Participant data and samples were collected between 2011 and 2021 at the Departments of Psychiatry and Neurology, University Clinic Ulm, Germany. All patients received a lumbar puncture during the diagnostic process. The patients or their legal representatives provided written informed consent to participate in the study, which aimed to identify diagnostic fluid biomarkers. Altogether, 208 patients were retrospectively enrolled: 100 with MDD, 25 with BD, 54 with SCZ, all diagnosed according to the ICD-10 system and 29 HCs. A summary of the demographic and neurochemical characteristics of the study groups is provided in table 1.

**Table 1:** Demographic characteristics and biomarker concentrations according to diagnostic group

	HC	MDD	BD	SCZ	P value
n	29	100	25	54	
Age (years)	46.5 (27–65)	45.0 (19–67)	43.3 (18–67)	34.4 (18–56)	**<0.0001** *
Sex (m/f)	9/20	40/60	13/12	32/22	*0.1090*
QAlb × 10^3^	4.2 (2.5–7.2)	5.2 (2.2–15.2)	6.1 (2.7–17.8)	4.9 (2.1–15.9)	**0.0084** **†**
BMI	n.d.	26.2 (18.3–43.6)	25.8 (17.9–39.3)	24.6 (15–34.4)	*0.3475*
SNAP-25 (pg/mL)	181 (66)	133 (44)	174 (69)	132 (59)	***0.0002* ‡**
NRXN3 (pg/mL)	151 (64)	111 (50)	122 (55)	105 (52)	*0.1162*
NRGN (pg/mL)	296 (130)	212 (77)	213 (107)	205 (116)	*0.2662*
NfL (pg/mL)	18 (8.7)	26 (27.4)	21 (17.1)	27 (68.8)	**0.0033§**
Aβ42 (pg/mL)	1292 (380)	916 (300)	1061 (251)	893 (293)	**0.0002¶**
Aβ40 (pg/mL)	12 351 (3190)	8971 (2599)	10 589 (2600)	8340 (2626)	**<0.0001** **
Aβ42/40	0.106 (0.006)	0.104 (0.022)	0.102 (0.019)	0.109 (0.025)	*0.2979*
ptau (pg/mL)	34 (9.7)	27 (8.5)	31 (9.2)	24 (9.0)	***0.0016* ††**
tau (pg/mL)	263 (91)	205 (69)	238 (89)	208 (104)	**0.0157‡‡**

Age, BMI and the albumin CSF–serum ratio (QAlb) is given as median (range) and biomarker concentrations as mean (SD). The p values result from the Kruskal-Wallis test (post-hoc results are given below) or χ2 test for sex distribution.

* HC vs SCZ p=0.0003; MDD vs SCZ p<0.0001; BD vs SCZ p=0.0178.

† HC vs MDD p=0.0407, HC vs BD p=0.0056.

‡ HC vs MDD p=0.0056; HC vs SCZ p=0.0033; MDD vs BD p=0.0413; BD vs SCZ p=0.0223.

§ MDD vs SCZ p=0.0019.

¶ HC vs MDD p=0.0010; HC vs SCZ p=0.0005.

** HC vs MDD p=0.0010; HC vs SCZ p=0.0001; BD vs SCZ p=0.0288.

†† HC vs MDD p=0.0335; HC vs SCZ p=0.0033.

‡‡ HC vs SCZ p=0.0038.

BD, bipolar disorder; BMI, body mass index; HCs, healthy controls; MDD, major depressive disorder; n.d., not determined; NfL, neurofilament light chain; NRGN, neurogranin ; NRXN3, neurexin-3; QAlb, albumin CSF–serum ratio; SCZ, schizophrenia; SNAP-25, synaptosomal-associated protein of 25 kDa.

The patients and controls underwent standardised clinical-psychiatric and clinical-neurological examinations. Participants with any signs of an inflammatory disease or with any clinical or imaging evidence for a neurodegenerative disease were excluded. Additionally, HC subjects showing any sign of a neuropsychiatric disorder were excluded. Furthermore, to exclude any patients with Alzheimer’s disease (AD) (co)pathology, those with ratios CSF Aβ42/40 <0.069 or Aβ42/ptau <8.1 were excluded. Using this approach, six patients were excluded (four SCZ, one BD, one MDD).

Within the MDD group, 37 patients were diagnosed with a moderate episode (26 recurrent), and 63 with a severe episode (46 recurrent); of the latter, 24 were diagnosed with additional psychotic symptoms (10 first episode, 14 recurrent). In 28 patients with MDD, severity was assessed using the Montgomery-Åsberg Depression Scale (MADRS). The exact antidepressant medication dosage administered to the patients at the time of lumbar puncture was known for 84 patients with MDD. Analysis was conducted using the fluoxetine dose equivalents.^9^ Similarly, the dosage of antipsychotic medication was known for 46 patients with SCZ and analysis was conducted using the olanzapine equivalents.^10^

### Sample collection and analysis

CSF was collected by lumbar puncture, centrifuged (1200 ×g, 10 min, RT), and within 2 hours was stored at −80°C until analysis. SNAP-25 was measured using a commercial kit on the Simoa HD-X platform (Quanterix, Billerica, USA). Aβ42, Aβ40, ptau (p181) and tau (total-tau) concentrations were determined with automated immunoassays using the lumipulse G600-II system (Fujirebio, Gent, Belgium) and NfL using the ProteinSimple Ella System (Bio-Techne, Minneapolis, USA). NGRN and NRXN3 were measured in subgroups dependent on sample availability (n=123 and n=59, respectively). For the analysis of NGRN, a commercial ELISA was used (Euroimmun, Lübeck, Germany).

NRXN3 was determined by a digital ELISA established in-house (Simoa, Quanterix, Massachusetts, USA), using a polyclonal antibody specifically recognising NRXN3 (AF5269, Biotechne, Minneapolis, Minnesota, USA). The capture antibody was coupled to paramagnetic beads at a concentration of 0.2 mg/mL. For detection, the antibody was biotinylated and diluted to a concentration of 0.6 µg/mL in 1% BSA, PBS, 0.05% Tween-20. Sample preparation included a 1:4 dilution with 1% BSA, PBS, 0.05% Tween-20 and heating up to 60°C for 30 min. We applied a two-step assay protocol: (1) incubation of the sample with the beads (250 000 functional and 250 000 helper beads) and the biotinylated antibody for 60 min (80 cadences); (2) incubation of the washed beads with streptavidin-β-galactosidase followed by addition of the substrate and automated imaging. For quantification, a standard curve with 1.56–100 pg/mL recombinant NRXN3 (5269-NX, R&D Systems, Minneapolis, Minnesota, USA) was used. The lower limit of quantification of the assay was 5.03 pg/mL.

All analyses were performed in a blinded manner in the neurochemical laboratory at the university hospital in Halle/Saale.

### Statistics

GraphPad Prism (V.8, GraphPad Software, La Jolla, USA) and IBM SPSS statistics (V.28) were used for analysis. Nonparametric tests were applied in all analyses. Two groups were analysed for significant differences using the Mann-Whitney test, and three or more groups were analysed using the Kruskal-Wallis test followed by post hoc Dunn tests in case of significant results. All p values reported are two-tailed. A potential confounding effect of group differences in patient age was analysed by ANCOVA with Bonferroni-corrected post hoc tests. Receiver operating characteristics curve analysis was used for calculation of sensitivity and specificity and for graphical visualisation of the effect of the variation in the cut-off values. The optimal cut-off level for dichotomising values was that which yielded the maximum value of the Youden index. To determine intermeasure correlations, Spearman rank coefficient was applied considering p<0.05 as significant.

## Results

### Demographic characteristics

We examined a cohort of 208 patients: 100 MDD, 25 BD, 54 SCZ and 29 HC. An overview of the demographic characteristics is given in table 1.

The MDD and HC groups were matched with respect to age (p=0.06603) and sex (χ*^2^* (2, 129) = 0.7671, p=0.3811). Patients with SCZ were younger than patients of any other diagnostic group, and sex distribution differed between diagnostic groups (χ*^2^* (4, 208) = 8.217, p=0.0417). The body mass index (BMI) did not differ between groups (p=0.3475). Some patients, namely 10% of MDD, 16% of BD and 13% of SCZ, had a mild blood–CSF barrier dysfunction; albumin CSF–serum ratio (QAlb) values were higher in patients with MDD and BD than in HCs (p=0.0407 and p=0.0056, respectively).

### Levels and correlation of synaptic proteins in the diagnostic groups

**Figure 1:**
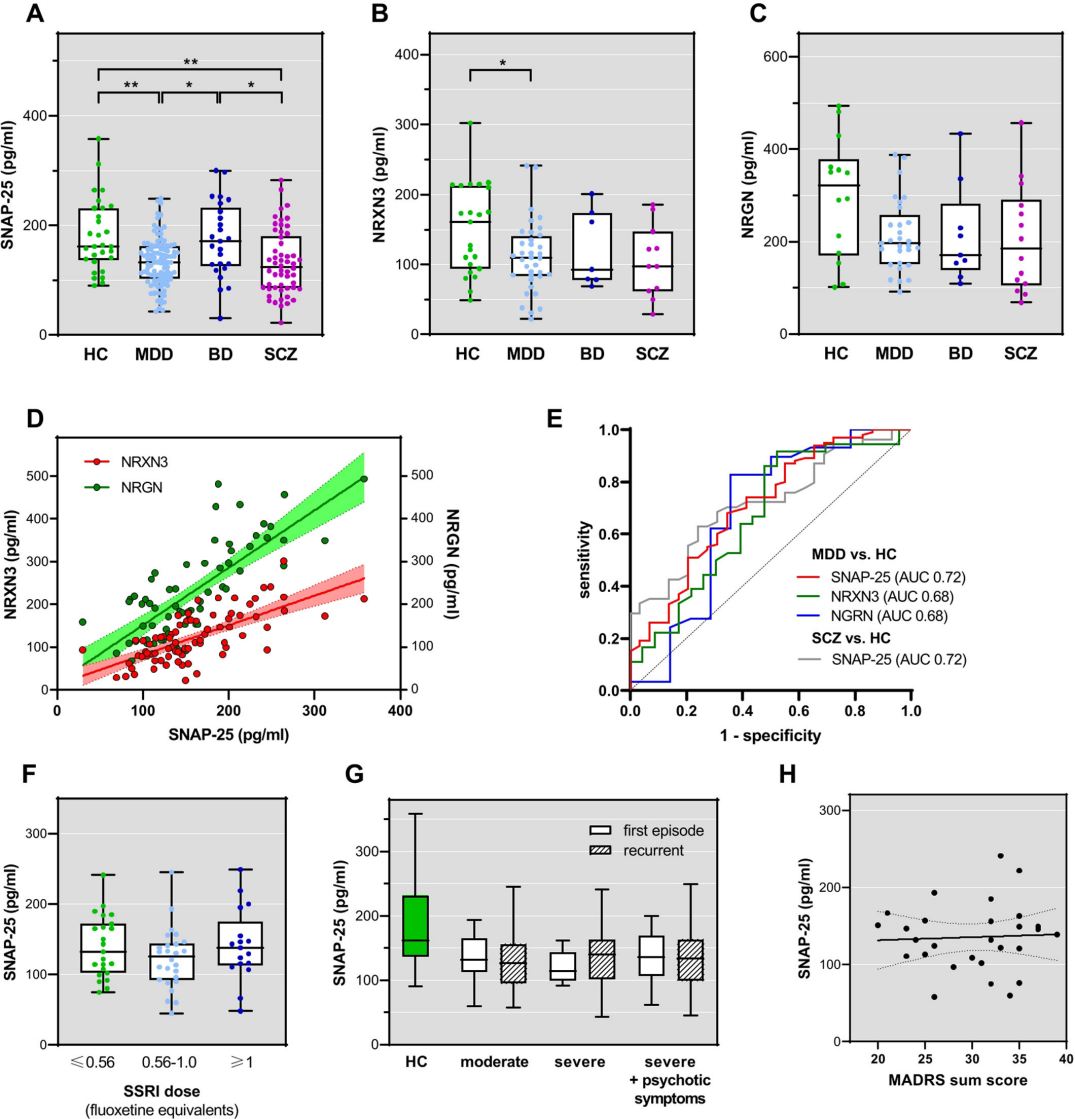
CSF levels of SNAP-25 (**A**), NRXN3 (**B**) and NRGN (**C**) according to diagnostic group. (**D**) Linear regression lines (with 95% confidence bands) for the inter-relationships between pairs of synaptic markers across all diagnostic groups. (**E**) Receiver operating characteristics curves for the discrimination of MDD versus HC based on SNAP-25, NRXN3 and NGRN, and for the discrimination of SCZ versus HC based on SNAP-25. Respective AUC values are given in the legend. (**F**) SNAP-25 in patients with MDD subgrouped according to the SSRI dosage at lumbar puncture. (**G**) Boxplots showing the SNAP-25 levels in patients with MDD categorised according to their ICD-10 diagnoses. Levels in HCs are shown for comparison. (**H**) Linear regression for SNAP-25 level versus MADRS sum score. Individual measures are presented with the linear regression line and 95% confidence bands. Box plots show the median, 25% and 75% percentiles, minimum and maximum whiskers, for synaptic protein concentrations. Asterisks indicate significant pair-wise differences (*<0.05, **<0.01) as determined by Kruskal-Wallis test and post-hoc testing. In B, the significant Mann-Whitney test result is indicated. AUC, area under the curve; BD, bipolar disorder; CSF, cerebrospinal fluid; HCs, healthy controls; MADRS, Montgomery-Åsberg Depression Scale; MDD, major depressive disorder; SCZ, schizophrenia; SNAP-25, synaptosomal-associated protein of 25 kDa; SSRI, selective serotonin reuptake inhibitors.

SNAP-25 levels were lower in patients with MDD compared with HCs (p=0.0004) and patients with BD (p=0.0416) (figure 1A). SNAP-25 levels were also lower in patients with SCZ than in HCs (p=0.0033) and BD (p=0.0223) and did not differ from those in patients with MDD. Including patient age as a covariate did not change these findings (HC vs SCZ: p=0.004, M_Diff_ 46.15, 95% CI (10.95, 81.34); HC vs MDD: (p<0.001, M_Diff_ 47.36, 95% CI (15.51, 78.21)).

NRXN3 and NGRN were on average lower in patients with MDD compared with HCs (figure 1B,C). While the overall analysis revealed no significant difference for the two markers (NRXN3 p=0.1162; NRGN p=0.2662), pairwise comparison of MDD versus HCs yielded a significant difference for NRXN3 (p=0.0268) and not for NRGN (p=0.0552).

When analysing all patients together, levels of SNAP-25, NRXN3 and NRGN were correlated robustly with each other (SNAP-25 and NRXN: r=0.70, SNAP-25 and NGRN: r=0.72, NRXN and NGRN: r=0.66; p<0.0001, respectively) (figure 1D). Diagnostic group-specific correlation analysis findings are given in table 2.

**Table 2:** Diagnostic group-dependent correlations between synaptic marker

	HC			MDD			BD			SCZ		
	N	r	P	N	r	P	N	r	P	N	r	P
SNAP-25–NRXN3	23	0.68	0.0003	36	0.53	0.001	7	0.68	0.110	10	0.88	0.002
SNAP-25–NGRN	14	0.67	0.01	29	0.60	0.001	7	0.63	0.076	13	0.75	0.005
NRXN3–NGRN	13	0.40	0.17	28	0.38	0.045	7	0.64	0.139	10	0.93	0.0003

Given are the Spearman correlation coefficients and the p values.

BD, bipolar disorder; MDD, major depressive disorder; SCZ, schizophrenia.

### Diagnostic sensitivity and specificity of CSF synaptic protein levels

Based on SNAP-25, MDD could be differentiated from HC with 68% (95% CI: 58% to 76%) sensitivity and 67% (95% CI: 47% to 80%) specificity (likelihood ratio 1.97) (figure 1E). NRXN3 reached a high sensitivity of 92% (95% CI: 78% to 97%) but obtained a low specificity of 48% (95% CI: 29% to 67%) (likelihood ratio 1.76). For NGRN, we calculated an 83% (95% CI: 66% to 92%) sensitivity and 64% specificity (95% CI: 39% to 84%) (likelihood ratio 2.32).

For the differentiation of SCZ from HC, SNAP-25 yielded 64% (95% CI: 50% to 75%) sensitivity and 77% (95% CI: 58% to 88%) specificity (likelihood ratio 2.61); NRXN3 reached 82% (95% CI: 52% to 97%) sensitivity and 52% (95% CI: 33% to 71%) specificity (likelihood ratio 1.71); and NGRN performed with 83% (95% CI: 52% to 92%) sensitivity and 64% (95% CI: 39% to 84%) specificity (likelihood ratio 2.32).

### Association of CSF synaptic protein levels with medication and MDD severity

The dosage of antidepressant medication was documented for 84 of the 100 patients with MDD. Comparing the SNAP-25 levels in MDD split into low, medium or high dosage of selective serotonin reuptake inhibitors (SSRIs) (figure 1F) or other antidepressants revealed no group differences (p=0.5753 and p=0.2848, respectively). Moreover, correlation and linear regression analyses revealed no connection between SNAP-25 levels and SSRI dosage (r=0.05989, p=0.5861; r^2^=0.0001, p=0.9160). Furthermore, there were no dosage-group differences for NRXN3 (p=0.7294 and p=0.1078, respectively) or NGRN (p=0.5677 and p=0.2867, respectively).

For 46 patients with SCZ, we could assess the association of SNAP-25 levels with the antipsychotic drug dosage expressed as olanzapine equivalents. Median-split analysis showed no association (p=0.3992). There was also no association between drug dosage and NRXN3 level (r=−0.3515, p=0.3519, n=9) or NGRN level (r=−0.0952, p=0.7691, n=12), but the small sample sizes should be noted.

Patients with MDD subgroup analysis for SNAP-25 levels was also conducted using ICD-10 MDD categories: MDD subgroups with moderate or severe episode, first episode or recurrent course of disorder, with or without additional psychotic symptoms could not be differentiated based on SNAP-25 (p=0.9580) (figure 1G). Analysis of the association of SNAP-25 levels with the MADRS score revealed no consistent inter-relationship (r=0.0381, p=0.8473) (figure 1H). There was also no evidence for an association with either ICD-10 subgroup or MADRS score for NRXN2 (p=0.4606) or NGRN (p=0.4934).

### Association of CSF synaptic protein levels with AD pathology and neurodegeneration markers

**Figure 2:**
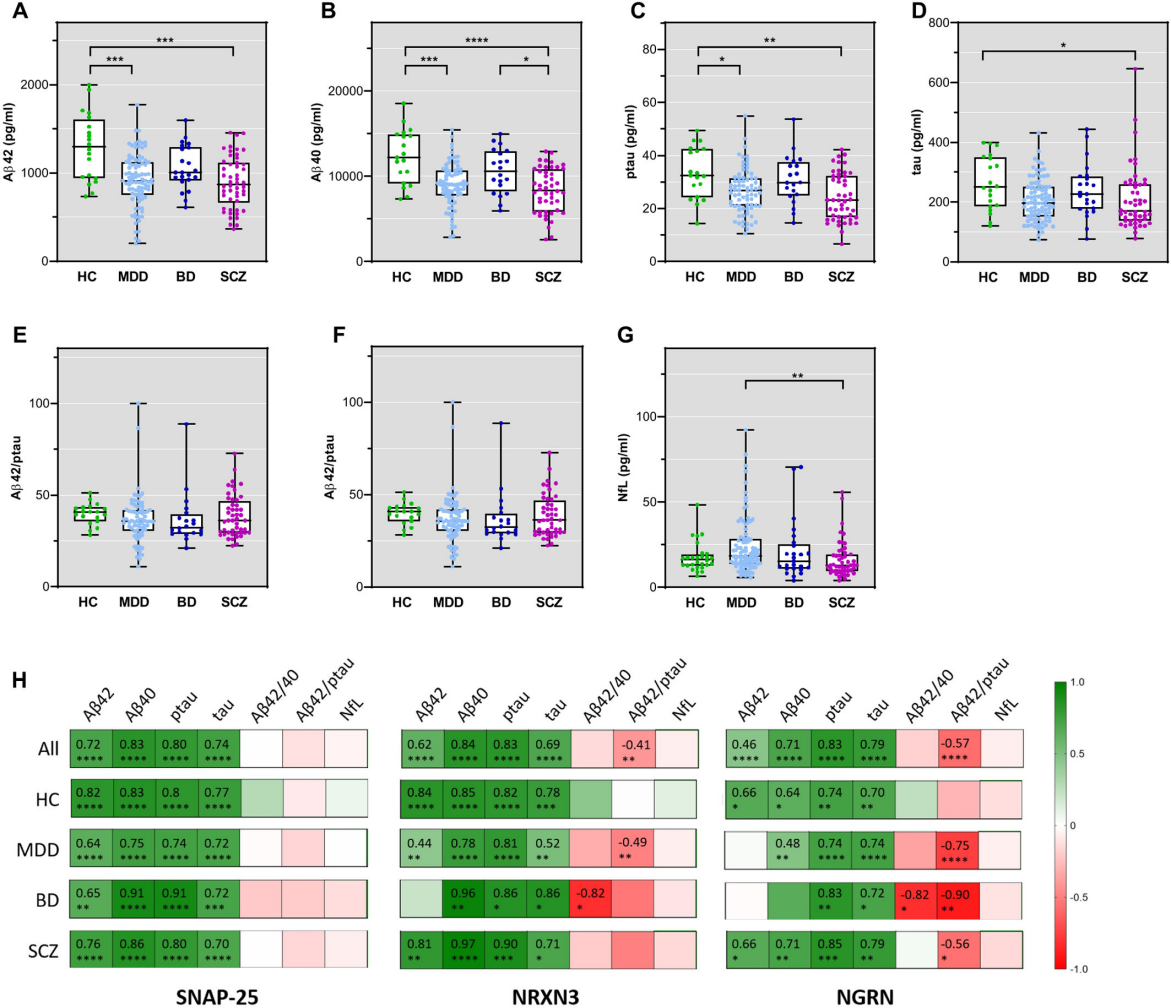
Diagnostic group levels of the Alzheimer panel markers Aβ42 (**A**), Aβ40 (**B**), ptau (**C**) and tau (**D**), the ratios Aβ42/40 (**E**) and Aβ42/ptau (**F**), and NfL (**G**). Box plots show the median, 25% and 75% percentile, minimum and maximum whisker concentrations. (**H**) Heat maps for the correlation between synaptic proteins, AD markers and serum NfL in the whole cohort and in diagnostic groups. Spearman correlation coefficients are colour-coded and given in the respective cells in case of significant results as indicated by asterisks (*<0.05, **<0.01, ***<0.001, ****<0.0001). AD, Alzheimer’s disease; BD, bipolar disorder; HCs, healthy controls; MDD, major depressive disorder; NfL, neurofilament light chain; NRGN, neurogranin; NRXN3, neurexin 3; SNAP-25, synaptosomal-associated protein of 25kDa; SCZ, schizophrenia.

Applying Mann-Whitney test, the CSF levels of Aβ42 (figure 2A) and Aβ40 (figure 2B) were lower in patients with MDD compared with HCs (p=0.0001 and p<0.0001, respectively). Also, ptau (figure 2C) and tau (figure 2D) were lower in MDD versus HCs (p=0.0032 and p=0.0080, respectively). The ratios Aβ42/40 (figure 2E) and Aβ42/ptau (figure 2F) were, however, comparable (p=0.2363 and p=0.0834, respectively). Reduced levels of the Alzheimer panel proteins were also observed for SCZ versus HCs (Aβ42: p=0.0005, Aβ40: p<0.0001, ptau: 0.0033, tau: p=0.0328, Aβ42/40 and Aβ42/ptau: p=<0.9999). For patients with BD, neither the levels of Aβ-peptides nor ptau or tau were significantly different from the levels in the other diagnostic groups, except for significantly higher Aβ40 levels compared with SCZ (p=0.0288).

Serum NfL differed between groups (p=0.0033) and post-hoc testing revealed higher levels in MDD compared with SCZ (p=0.0019) (figure 2G). Compared with HC, NfL levels were not different in any diagnostic group (MDD: p=0.285, BD: p>0.9999, SCZ: p=0.7638).

SNAP-25 levels were strongly correlated with the levels of Aβ42, Aβ40, ptau and tau in each diagnostic group (figure 2H, left panel). Similar patterns were seen for NRXN3 and NRGN and the Alzheimer panel proteins. With respect to an association with the ratios Aβ42/40 and Aβ42/ptau, we observed differences between the synaptic markers: while SNAP-25 was not associated with the ratios as mentioned above, NRXN3 and NGRN showed a moderate correlation with Aβ42/ptau in the whole cohort and in MDD specifically. Additionally, NGRN was associated with Aβ42/ptau in BD and SCZ. For Aβ42/40, association was obtained with NRXN3 and NRGN in patients with BD specifically.

## Discussion

The main finding of our study is that SNAP-25 is reduced in the CSF of patients with MDD and therefore has potential as a diagnostic marker. We also provide first evidence that SNAP-25 is reduced in SCZ. Furthermore, correlations between CSF levels of SNAP-25 and AD pathology markers were found to show subtle differences between psychiatric disorders.

Alterations in a variety of molecular mechanisms with a complex interplay are proposed to underlie MDD, with one major hypothesis being that these pathophysiological changes converge at synapses.^11^ Compartmental biomarkers that reflect synaptic protein changes in the brains of patients with MDD might help to elucidate the underlying pathophysiological processes and support diagnosis and the ascertaining of disorder severity. Moreover, treatment-resistant patients could potentially be detected prior to antidepressant therapy or treatment effects could be monitored by means of fluid biomarkers in clinical trials that target synaptic function. In our previous study, we detected a biosignature of decreased synaptic proteins in the CSF of MDD^6^ corroborating earlier genetic findings.^4^ Furthermore, the SNAP-25 expression pattern is altered in MDD brains.^12^ Here we hypothesised that SNAP-25, specifically, is changed in the CSF of patients with MDD reflecting synaptic dysfunction.

Studying SNAP-25 expression in CSF samples of a large cohort of clinically well-characterised MDD patients, we observed significantly decreased levels compared with control subjects without signs of mental illness, inflammation or neurodegeneration. Lower CSF levels could reflect reduced SNAP-25 expression in intact presynaptic terminals, reduced synaptic densities or both. Changes in the synaptic expression levels have been reported for a variety of neurological and neuropsychiatric diseases and conditions.^6 7^ Furthermore, inflammatory conditions are associated with SNAP-25 dysregulation, for example, in autoantibody-mediated encephalitis. Here, the internalisation of synaptic membrane receptors is proposed to induce reduction of SNAP-25 at the synapse resulting in decreased SNAP-25 levels in the CSF.^13^ In neurodegenerative diseases, the increase of SNAP-25 is mostly supposed to be based on synaptic degeneration and loss which is then reflected by increased CSF levels.^7^ Importantly, for both synaptic dysregulation and atrophy, there is evidence that SNAP-25 CSF levels can be used to mirror disorder severity.^13 14^

For MDD, there is evidence from postmortem studies for reduced SNAP-25 expression in hippocampus areas and for reduced expression of synapse-related genes and reduction in synapse density in prefrontal cortex.^4^ Recently, based on neuroimaging data, the reduction in synaptic density in late-life depression was questioned.^15^ Furthermore, synaptic loss has been proposed to lead to increased rather than decreased brain SNAP-25 levels in neurodegenerative diseases.^8^ Therefore, we propose that CSF SNAP-25 level might represent an integrated readout of reduced synaptic function, rather than of synaptic degeneration, in MDD.

It is important to know the effect of antidepressant treatment on the status of a potential MDD biomarker, as most patients already begin antidepressant therapy prior to seeing a specialist and the opportunity for CSF sampling. In a rodent chronic stress model, chronic fluoxetine treatment changed the synaptoproteome of the prefrontal cortex.^16^ Here, SNAP-25 levels in the CSF did not correlate with the SSRI dosage that the patients received at the time of lumbar puncture, providing first evidence for the validity of CSF SNAP-25 as an MDD biomarker. Also, we did not observe differences in the SNAP-25 CSF level according to the MDD course or its severity. Therefore, CSF SNAP-25 might be used in first episodes or in recurrent disorder as well as in patients with moderate to severe depressive symptoms, for example, as read-out in therapeutic trials.

To study the potential utility of SNAP-25 for the differential diagnosis of mood disorders, we analysed BD and SCZ CSF samples as well. While BD showed biomarker levels in the range of HC, SCZ was characterised by decreased SNAP-25 levels. This might open a discussion on similar molecular effects in MDD and SCZ, whereas BD is surprisingly different. Synaptic pathology and changes in vesicle trafficking and synaptic protein levels were attributed to BD and SCZ, with findings in part divergent dependent on the brain region examined.^17^,^19^ Pilot studies applying quantitative immunoblot to CSF samples found SNAP-25 to be increased in SCZ and unchanged in BD,^20 21^ in line with current findings, while levels in first episode psychosis were unchanged.^22^ Multicentre studies of well-characterised SCZ subjects are required to attempt to validate the current findings, which suggest potential diagnostic applicability of SNAP-25 in SCZ.

CSF expression studies of synaptic proteins in primary psychiatric disorders are scarce but could elucidate pathophysiological changes at synapses. A variety of synaptic proteins were found to be downregulated in MDD and SCZ; this includes NRXN3^4 6 23^ which we could confirm using a newly established Simoa assay. Postsynaptic NGRN was unchanged in late-life MDD,^24^ BD^25^ and first episode psychosis,^26^ which we could corroborate with this quite comprehensive study. The trend for decreased levels of all three examined synaptic biomarkers and the high association between their levels may reflect a general reduction in synaptic expression or activity, rather than synaptic, dendritic or axonal degeneration. Accordingly, NfL is not increased in MDD,^27^ whereas a variety of studies on neurodegenerative diseases such as AD showed increased synaptic proteins in the CSF.^8^ This idea is supported by our observation of strong correlations between synaptic and Alzheimer markers. Interestingly, we could also observe alterations in ptau, tau, Aβ42 and Aβ40 in patients with MDD, although the values were in the physiological range. This might point to a subtle neurodegenerative change, which only becomes evident when looking at the synaptic protein levels. Such correlations have been previously reported for healthy elderly^28^ and for patients of the AD continuum.^29^ The Alzheimer protein panel changes were interpreted as evidence for molecular dysfunction in synaptic processes which potentially drive early Alzheimer pathological changes,^28^ or vice versa.^30^ Future studies covering the spectrum of psychiatric towards neurodegenerative diseases will help to elucidate the inter-relationship between synaptic proteins and established AD pathology markers and the significance of subtle differences in the strength of association. To determine both the diagnosis and the stage dependence of synaptic biomarker changes, longitudinal studies will also be important. As the first blood assays for synaptic markers recently became available, compilation of large multicentre cohorts will set the basis to further study the significance and diagnostic potential of SNAP-25 and other synaptic proteins.

The strength of our study is that we were able to investigate a large cohort of psychiatric patients and controls and that other disorders such as organic psychiatric disorders could be excluded by state-of-the-art neuroimaging. However, our study has some limitations: NRXN3 and NRGN could not be measured in all patients resulting in some small sample sizes for the subgroup analyses, for example, correlation analyses with medication. Furthermore, the MADRS sum score was reported for only one third of our patients with MDD. The important potential confounders, drug/substance abuse and duration of antidepressant therapy, could not be regarded in our study. A prospective design of future studies with larger sample sizes of a diverse population and with consideration of a broader spectrum of potential confounders is crucial to reach a better validity of results.

We conclude that synaptic molecular dysfunction and/or synaptic degeneration can be reflected in CSF protein expression changes. We now provide evidence for reduced CSF levels of SNAP-25 in MDD and SCZ, with subtle differences in the associations among markers which encourages further examination of the relationship with diagnosis, staging and therapy effects.

## Data Availability

Data are available upon reasonable request.
